# The Proximate Composition, Mineral and Pectin Content and Fatty Acid Profile of the Pomace Fraction of 16 Rowanberry Cultivars

**DOI:** 10.3390/plants13121615

**Published:** 2024-06-11

**Authors:** Viive Sarv, Shehzad Hussain, Reelika Rätsep, Ave Kikas

**Affiliations:** 1 Polli Horticultural Research Centre, Chair of Horticulture, Institute of Agricultural and Environmental Sciences, Estonian University of Life Sciences, Uus 2, 69108 Polli, Estonia; reelika.ratsep@emu.ee (R.R.); ave.kikas@emu.ee (A.K.); 2 ERA Chair for Food (By-) Products Valorisation Technologies (VALORTECH), Estonian University of Life Sciences, Fr. R. Kreutzwaldi 56/5, 51006 Tartu, Estonia; shehzad.hussain@emu.ee

**Keywords:** rowanberry cultivars, dietary fiber, pectin, minerals, fatty acids

## Abstract

The berry pomace could be a potential source for food applications due to its high content of polyphenols, but also dietary fiber, PUFAs and pectin. This is the first study that aims to compare the total dietary fiber (TDF), protein, fat, mineral, pectin and fatty acid content of the following 16 different pomace samples of *Sorbus aucuparia* L. cultivars (cvs): ‘Likernaja’, ‘Burka’, ‘Alaja Krupnaja’, ‘Granatnaja’, ‘Rubinovaja’, ‘Bussinka’, ‘Vefed’, ‘Angri’, ‘Krasnaja’, ‘Solnechnaja’, ‘Sahharnaja’, ‘Oranzevaja’, ‘Kubovaja’, ‘Moravica’, ‘Rosina’ and ‘Rossica’, in order to find new natural materials for valorization. The contents of pectin and dietary fibers were analyzed using the respective Megazyme enzymatic kits. The TDF content was the highest in the pomace samples of hybrid cvs ‘Granatnaja’ (63.04% dry mass DM), ‘Burka’ (64.52% DM), ‘Rubinovaja’ (65.66% DM) and ‘Likernaja’ (67.17% DM). The pomace of hybrid cv ‘Rubinovaja’ was distinguished from other samples by its high protein content, cv ‘Alaja Krupnaja’ by its high pectin content and cv ‘Oranzevaja’ by its high fat content, which were 7.58% DM, 8.39% DM and 7.47% DM, respectively. The pomace of cv ‘Sahharnaja’ possessed the highest average macro-element content (1.56 g/kg DM). The average fatty acids profile was characterized by a high content of linoleic acid (51.94%), oleic acid (20.55%) and palmitic acid (12.96%). The lowest n6/n3 ratio was found in the hybrid cv ‘Alaja Krupnaja’ (6.70%). The data obtained demonstrate that the pomaces of certain cultivars of rowanberry contain significant amounts of valuable components, which can be used in functional food and cosmetic applications.

## 1. Introduction

Juice production generates a remarkable amount of fruit by-product, called pomace, which consists of various berry components, such as skins, seeds and pulp. These fruit parts are rich in bioactive polyphenols, dietary fiber, polyunsaturated fatty acids, minerals, proteins and other components [1]. According to numerous scientific publications, most of these compounds possess health-promoting effects, such as antioxidant, anti-inflammatory, anti-diarrheal, anti-diabetic, antitumor, as well as vasodilatory and diuretic activity [2,3]. However, despite the considerable number of valuable components, the majority of fruit pomace is still discarded in landfills and is not used for the benefit of human health. According to the current goals set by the Food and Agriculture Organization of the United Nations (FAO) for the 2030 agenda, juice production must substantially reduce the generation of discarded by-products [4] and, therefore, it is essential to find opportunities for their biorefinement.

The dietary fiber forms the main part of berry pomace. The incorporation of plant-based dietary fibers into food products can provide various functional properties to food matrices, for instance, enhancing their water- and oil-adsorption capacity, their swelling ability and their antioxidant capacity [5]. In addition, dietary fibers can improve colonic health by protecting cells against oxidative stressors [3]. Although many experts have suggested a total dietary fiber (TDF) intake of 25 to 30 g per day with 6 to 8 g per day of soluble fiber, the global consumption of dietary fiber is still less than 20 g per day per person [6]. TDF includes several organic compounds, such as cellulose, hemicelluloses, pectin, hydrocolloids and lignin, of which the hemicelluloses, cellulose and lignin are resistant to human digestive enzymes [7]. The crude fiber (CF) determination approach is a fairly simple method used for the proximate estimation of indigestible fiber in plant material, used for characterizing not only animal feed, but also agronomic by-products. CF measures cellulose, pentose and lignin in the sample, but does not determine hemicelluloses, pectin and hydrocolloids, because they are digested by the dilute acid and alkali in the determination process and are therefore not collected. For the determination of TDF, the defatted food sample is treated with enzymes that mimic the digestive process in the human small intestine. In order to mimic the absorption of sugars in the body, the digestible carbohydrates are broken down into simple sugars and removed from the sample by precipitation and filtration. The non-digestible precipitate contains dietary fiber as well as protein and inorganic material, which must be measured in a separate process and subtracted from the weight, to calculate the TDF content. TDF is divided into water-insoluble (or least-fermented) dietary fiber (IDF), such as cellulose, hemicellulose and lignin, and water-soluble dietary fiber (SDF), such as pentosans, pectins, gums and mucilage. Protein in the TDF sample is digested with protease. For the measurement of IDF, the digestate is filtered and the IDF is determined gravimetrically. The SDF is precipitated by adding ethanol to the filtrate of the IDF. The precipitated SDF is filtrated and determined gravimetrically after correction for any protein or ash in the precipitate. As these procedures are more sophisticated, involving various enzymes, the CF assay is still in use for the proximate estimation of indigestible fiber.

IDF influences food texture and improves intestinal passage time, while SDF influences the levels of whole cholesterol and low-density lipoprotein cholesterol in the serum [8]. Berry pectins, which are considered an important source of soluble fiber, represent heteropolysaccharides with a variable molecular mass, and their backbone is mainly composed of galacturonic acid residues bonded via α-(1→4) glycosidic linkages [9]. According to the European Food Safety Authority (EFSA), pectin has been recognized as a nutritional supplement used for the purpose of reducing the glucose response, low-density lipoprotein cholesterol and the levels of serum total cholesterol [10]. In order to achieve the maximum health benefits from fibers during the fortification of foods, the sources of dietary fibers should have a IDF/SDF ratio between 1:1 and 2:1 [9]. Although the IDF/SDF ratio in fruit pomace widely exceeds 2:1, the pomace fiber can act as a carrier of phenolic compounds with antioxidant effects and, therefore, can be considered to be an antioxidant dietary fiber [11].

In addition to dietary fiber, fruit pomace contains a seed fraction, which is considered to be a valuable source of bioactive phytochemicals, including fatty acids. Various types of fatty acids can be classified according to their length, as well as the number and position of double bonds in the hydrocarbon chain, while the existence of double bonds specifies the degree of unsaturation in fatty acids. Essential fatty acids (EFAs) include omega-3 (ω-3) and omega-6 (ω-6) fats, which are important for human brain function and cell growth [12]. Moreover, several studies have proven the positive effect of EFAs originating from fruit seed oil for their ability to prevent or treat diseases, such as cancer [13], hyperlipidemia [14] or cardiovascular diseases [15]. Since the human body itself does not synthesize EFAs, they can only be obtained from food [16]. Several preparations obtained from blackberry, blueberry, raspberry and strawberry seed oils are already available on the market; however, there are still many less-investigated berry oils, which could be relevant sources for functional foods [17,18] or cosmetics [19]. In order to know the characteristics of scarcely investigated fruit sources, it is essential to analyze their specific qualitative and quantitative composition.

Rowanberries (*Sorbus aucuparia* L., *Rosaceae* family) are mainly grown as decorative trees or shrubs in parks and gardens in the Northern Hemisphere. In terms of their applications as foods or nutraceuticals, rowanberries are still considered rather underutilized plants. The orange rowanberry fruits have traditionally been used as anti-inflammatory, laxative or diuretic agents, as well as treatments for kidney diseases and various gastrointestinal disorders [20]. Despite their astringent flavor, rowanberries have also been used for juice, wines or jams [21]. The rowanberry juice pressing generates 15–20% rowanberry pomace, which due to its high content of valuable components [22,23] can be valorized as functional ingredients for food and other relevant uses. To obtain more palatable varieties of rowanberries, in the 19th century the sweeter clones of *S. aucuparia* L. from the Sudety Mountains (the Moravian Mountain Ash) were selected for breeding. At the beginning of the 20th century, a breeding program was started by Russian plant breeder Michurin, resulting in interspecific hybridization with *S. aucuparia* L. with *Malus*, *Aronia*, *Pyrus*, and *Crataegus* species [24]. Data on cultivar-specific rowanberry press residues, such as their proximate composition, macro- and microelement content, pectin content, and fatty acid composition, have not yet been published.

In a previous study, the composition of phenolic compounds, such as phenolic acids, flavonoids, and anthocyanins of 16 sweet rowanberry cvs, as well as their antioxidant properties and breeding background, were evaluated [23]. In the current study, for the first time, the proximate composition, mineral and pectin content and fatty acid profile of pomace fractions of rowanberry pomace samples of five hybrid cvs ‘Likernaja’, ‘Burka’, ‘Alaja Krupnaja’, ‘Granatnaja’ and ‘Rubinovaja’, as well as eleven sweet rowanberry cvs ‘Bussinka’, ‘Vefed’, ‘Angri’, ‘Krasnaja’, ‘Solnechnaja’, ‘Sahharnaja’, ‘Oranzevaja’, ‘Kubovaja’, ‘Moravica’, ‘Rosina’ and ‘Rossica’, were evaluated in order to understand their chemical composition on a cultivar basis and their potential suitability for food applications.

## 2. Results and Discussion

### 2.1. The Proximate Composition

The proximate composition of pomace samples of 16 rowanberry cvs is presented in Table 1. The average moisture content of lyophilized pomace was 2.45% (Table 1). It appears that most of the rowanberry pomace consisted of dietary fiber, ranging from 52.71% DM for cv ‘Oranzevaja’ to 67.17% DM for cv ‘Likernaja’. The mean CF concentration of rowanberry pomace samples of 16 cvs was 15.64% DM, which formed approximately 25% of the mean TDF concentration. Both TDF and CF contents were the highest in the dry pomace samples of hybrid cvs (‘Granatnaja’, ‘Burka’, ‘Rubinovaja’ and ‘Likernaja’), while the contents in ‘Likernaja’ 67.17% DM for TDF and 21.19% DM for CF, exceeded the others. Bobinaitė et al. analyzed the CF content of an industrial rowanberry pomace mixture, obtaining a result of 19.57% DM [22], which is similar to the average CF value (19.51% DM) of pomace from four hybrid cvs (‘Granatnaja’, ‘Burka’, ‘Rubinovaja’ and ‘Likernaja’) obtained in this research. In addition, Reißner et al. analyzed the TDF content in rowanberry pomace samples (660 g kg^−1^) [25], which was similar to the average TDF value of four hybrid cvs (65.10% DM) in the current study. Moreover, Reißner et al. found that the content of TDF in rowanberry pomace was higher than the TDF content in blackcurrant, redcurrant, gooseberry, or chokeberry pomace [25]. Alba et al. claimed that fruit-based dietary fiber supplies physiological and technological advantages as well as preferable nutritional value compared to other sources of dietary fiber, due to the lower IDF/SDF ratio [9]. According to Reißner et al. the IDF/ SDF ratio is 7.75 in rowanberry pomace [25]. Similar IDF/SDF ratios have been found in red currant (7.30), gooseberry (7.04), chokeberry (7.45), blueberry (6.56), bilberry (7.54) [11] and lingonberry (7.70) [5], while in raspberry, cranberry, blackcurrant and sea buckthorn the ratios were higher 24.87, 11.49 and 13.89 [11] and 11.93 [5], respectively. According to Reißner et al. the high content of TDF might be a result of remaining pectin in rowanberry pomace because, unlike other berries, the rowanberries were not enzymatically treated before pressing [25].

Commercially, pectin has been extracted from apple pomace (up to 14%) and from peel of citrus fruits (85%). However, researchers have been recently looking for options to extract natural pectin from agro-industrial by-products, such as fruit pomace [26] in order to valorize agro-industrial wastes. Moreover, since both apple and rowanberry belong to the same subtribe *Malinae*, it was hypothesized that similarly to apple pomace, rowanberry pomace could be a good source of pectin. In the current study, the pectin content in 16 rowanberry pomace samples was the highest (8.39% DM) in the hybrid cv ‘Alaja Krupnaja’ (Table 1) and the average value of all cvs was 6.87% DM. Previously, Zlobin et al. determined a slightly lower value of pectic polysaccharides 4.2% DM [27]; however, Tańska et al. demonstrated a 4-fold lower content of pectins (1.1% DM) in rowanberry pomace [28]. In their study, the pectin content was slightly higher in rosehip, blackcurrant, and elderberry pomace: 3.7% DM, 1.8% DM and 1.8% DM, respectively [28]. 

The protein and fat in fruit pomace samples originate mainly from the seeds. The pomace of sweet rowanberry cv ‘Rubinovaja’ possessed the highest protein concentration in the current study (7.58% DM), which was similar to the value of 7.09% DM in the study by Reißner et al. [25]. However, the average protein concentration of 16 pomace samples in the current study was only 4.60% DM, which was lower than in most previously analyzed fruit pomaces, such as blueberry (6.64% DM) [29], cranberry (7.4% DM), lingonberry (8.60% DM) [5], apple (6.91% DM), strawberry (16.22% DM), black currant (12.76% DM), and chokeberry (10.77% DM) [30]. The highest fat concentration (7.47% DM) was measured in cv ‘Oranzevaja’ pomace. The average fat content of 16 samples was 5.68% DM, which was 1.4-fold higher than the 3.97% DM value detected by Reißner et al. [25]. Comparing the fat content in the rowanberry pomace with the fat content in the pomace samples of various other berries revealed that blueberry (4.05% DM) [29], apple (3.30% DM) and chokeberry (5.15% DM) [30] pomace samples had fat contents more similar to the rowanberry pomace than cranberry (9.83% DM), lingonberry (12.68% DM) [5], strawberry (11.63% DM) and blackcurrant (10.52% DM) [30] pomace samples. Although the data on the chemical composition of rowanberry pomace in the literature are scarce, the studies by Reißner et al. [25], Bobinaitė et al. [22], Zlobin et al. [27] and Tańska et al. [28] provide an opportunity to compare the composition of rowanberry pomace from various growing locations with the rowanberry pomace composition collected from Polli Horticultural Research Centre, South Estonia. Reißner et al. published a comprehensive overview of the proximate composition (moisture, ash, fiber, protein, fat content) of various berry pomace powders and their technofunctional properties (seed content, particle size, water activity, water binding and fat absorption capacity, swelling capacity). However, the current study focused on the peculiarities in the composition of different rowanberry cvs. In addition to their proximate composition, their macro- and microelement content, pectin content, and fatty acid profile were also investigated. The discrepancies in the results of different authors presumably depend on the different determination methods, the variations in the cultivation, cultivars, maturity, and processing and growing conditions of the rowanberries. For instance, Zlobin et al. obtained the rowanberries (*Sorbus aucuparia*) grown in Kirov, Russia; Tańska et al. in Olsztyn, Poland; Reißner et al. in Eastern Germany, and Bobinaitė et al. obtained the pomace from Lithuanian food industry company. According to Köppen climate classification [31] Polli Horticultural Research Centre in Estonia is situated in the temperate climate zone between maritime and continental climate, characterized by warm summers and fairly mild winters. Kirov in Russia has humid continental climate, with warm and rainy summers and cold and extremely snowy winters. Eastern Germany has hot summers and cold winters, and Olsztyn, Poland is characterized by oceanic climate, while North Eastern Anatolia region of Turkey corresponds to Central European weather conditions with a quite cold and wet climate throughout the year.

### 2.2. The Macro- and Microelement Contents

**Figure 1:**
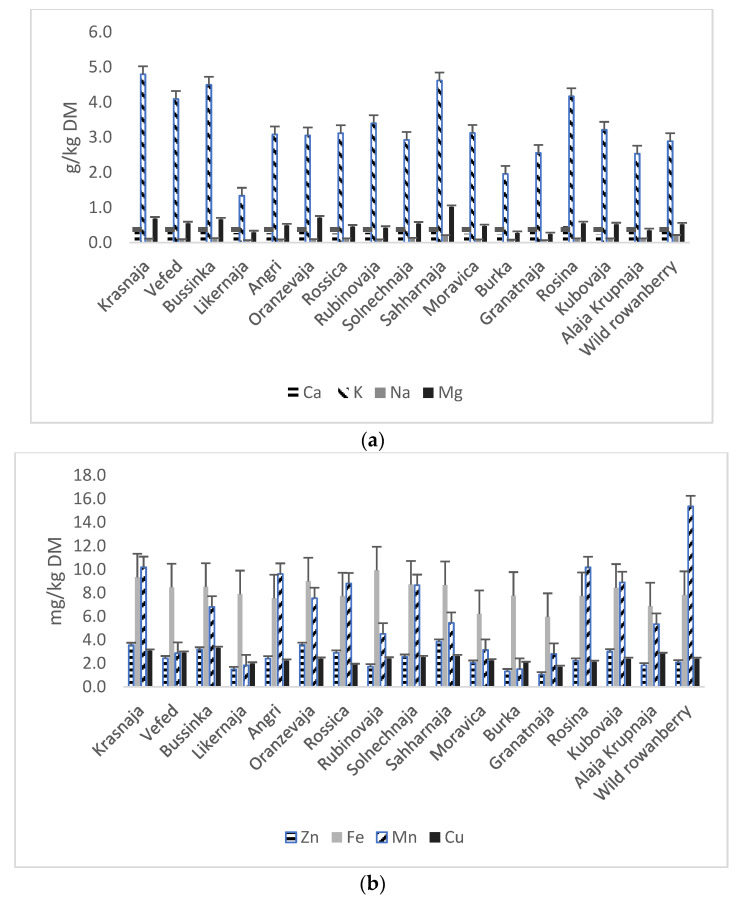
The macro- (**a**) and microelements (**b**) in pomace samples of 16 rowanberry cvs and wild rowanberry.

According to the European Society for Clinical Nutrition and Metabolism (ESPEN) recommendations for microelement intakes (2022) for 31–70 year-old healthy people, copper (Cu), iron (Fe), manganese (Mn), and zinc (Zn) should be provided in dosages of 0.9, 8, 1.8–2.3 and 8–11 mg/day, respectively [32].The macro- and microelement contents of the examined 16 rowanberry pomace samples and a wild rowanberry pomace sample are given in Figure 1. The pomace of sweet rowanberry cv ‘Sahharnaja’ stood out from other rowanberry pomace samples for the highest content of magnesium (Mg, 1.01 g/kg DM) and Zn (3.8 mg/kg DM), as well as the highest average macro-element (Ca, K, Na, Mg) content (1.56 g/kg DM), while the pomace of cv ‘Rosina’ had the highest calcium content (Ca, 0.46 g/kg DM) (Figure 1). The pomace of wild rowanberry had the highest sodium (Na, 0.21 g/kg DM) and Mn (15.4 mg/kg DM) content, as well as the highest average microelement (Zn, Fe, Mn, Cu) content (6.9 mg/kg DM). The hybrid cv ‘Rubinovaja’ differed from the other pomace samples for the highest content of Fe and cv ‘Bussinka’ for the highest content of Cu, 9.92 mg/kg DM and 3.31 mg/kg DM, respectively. The most dominant macro-element in rowanberry cvs is potassium, with average value of 3.66 g/kg DM and the highest value of 4.79 g/kg DM in cv ‘Krasnaja’ pomace. The average iron content (8.03 mg/kg DM) was the highest among microelements, while the highest value (9.92 mg/kg DM) was found in hybrid cv ‘Rubinovaja’. 

In the study by Aslantas et al., where the macro- and microelement contents of various wild fruit species were evaluated [33], the potassium content in rowanberries was found to be 1.54 g/kg DM, which was almost 2-fold lower than the potassium content in wild rowanberry pomace in the current study. Similarly, Aslantas et al. reported a 1.7-fold lower magnesium content (0.29 g/kg) in rowanberry pomace than in the current study (0.50 g/kg DM). On the other hand, the study by Aslantas et al. [33] stated the high content of phosphorus in wild fruit species, while the phosphorus content in rowanberry pomace samples in the current study was only marginal (less than 30 µg/kg DM on average). The rowanberries (*Sorbus aucuparia*) analyzed by Aslantas et al. were naturally grown in Northeastern Anatolia, Turkey. 

Similarly to rowanberry pomace, potassium is the most prevalent macro-element in apple and black currant pomace. Iron was the most abundant microelement in all investigated fruit pomace samples, such as in apples, strawberries, back currants, and chokeberries, according to the study by Pieszka et al. [30]. Moreover, Sójka et al. [34] similarly found that iron was the most prevalent microelement in chokeberry pomace; however, with a significantly higher value than in rowanberry pomace (78.6 mg/kg DM). In addition, unlike the rowanberry pomace, calcium (4.08 g/kg DM) was the most abundant macro-element in dried chokeberry pomace. In the study by Sójka et al. [34], the average magnesium content was 1.6-fold lower than the calcium content, while in the case of rowanberry pomace in the current study, the calcium/magnesium ratio was visa versa: the average calcium content (0.31 g/kg DM) versus magnesium content (0.50 g/kg DM). 

The study by Sójka et al. [34] revealed that, generally, the seed fractions contain up to 10-fold more magnesium than seedless fractions, while calcium and sodium are present mainly in juice and seedless fractions. Moreover, zinc and copper were more prevalent in seed fractions, while iron existed only in seedless fractions [34]. These findings should be confirmed for other fruits, such as rowanberries, in the future. However, the current study proved the high content of potassium, manganese, iron, and other macro-and microelements in rowanberry pomace. Therefore, considering 1.54 mg Mn/100 g in wild rowanberry sample, as well as the significant content of other microelements (Zn, Fe, Cu) with an average of 0.4 mg/100 g and the Mn requirements for the human diet (1.8–2.3 mg/day), the wild rowanberry pomace can be used as a potential fiber- and microelement-rich food ingredient to make flake-type snacks. 

### 2.3. The Content of Fatty Acids

**Figure 2:**
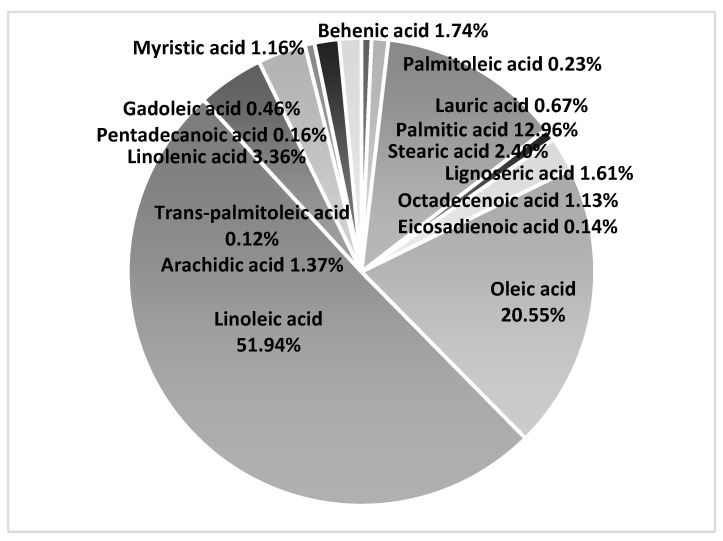
The average concentrations (%) of individual fatty acids (based on total fatty acids) in the pomace samples of 16 rowanberry cultivars.

The fatty acids composition in dried pomace of 16 rowanberry cvs was characterized by a high content of linoleic acid (C18:2), oleic acid (C18:1), and palmitic acid (C16:0), constituting on average 51.94%, 20.55%, and 12.96%, respectively (Figure 2). The high contents of linoleic, oleic, and palmitic acids in rowanberry pomace are consistent with the previously determined results of Bobinatė et al.; however, in their study, the contents of linoleic and oleic acids were higher, 61.18% and 22.25%, respectively, and palmitic acid content was lower (9.00%) [22]. Bobinatė et al. obtained the frozen rowanberry pomace mixture from the Lithuanian joint-stock company, while in the current study, the pomace was made on site by pressing the juice separately from every rowanberry cultivar using a low-speed juicer. Among the other analyzed fatty acids in the current study, only four (linolenic, arachidic, stearic and behenic acid) exceeded 1% of all fatty acids, ranging up to 3.36%, 1.37%, 2.40%, and 1.74%, respectively (Figure 2).

Comparing the fatty acid composition in pomace samples of different rowanberry cultivars, it was revealed that the highest total fatty acid content per sample was found in cvs ‘Sahharnaja’ and ‘Oranzevaja’, 4.69%and 4.52%, respectively, which correlated with the highest crude fat content of the same cvs (Table 1 and Table 2). Moreover, the same cvs contained the highest contents of linoleic acid, 57.33% and 57.69%, respectively, of all fatty acids in pomace. The highest level of α-linolenic acid was found in hybrid cvs ‘Burka’, ‘Granatnaja’, and ‘Alaja Krupnaja’, with ‘Alaja Krupnaja’ exceeding the others with a value 6.08%. Both linoleic acid and α-linolenic acid are the only essential fatty acids for humans. All the cvs, except the hybrid ones had an oleic acid (monounsaturated fatty acid) content higher than 20%, while the level of palmitic acid (saturated fatty acid) exceeded 20% only in two hybrid cvs ‘Alaja Krupnaja’ and ‘Granatnaja’.

Pieszka et al., who investigated the fatty acids composition of dried pomaces of apples, strawberries, black currants, and chokeberries, found the highest level of linoleic acid in apples (64.35%) [30]. In strawberries, black currants, and chokeberries, the level was lower than the average level of linoleic acid in dried pomace of 16 rowanberry cvs (51.94%). Moreover, in their study, the strawberry and chokeberry pomaces contained high levels of oleic acid (monounsaturated fatty acid): 17.36% and 16.38% [30], respectively, similar to rowanberry pomace (20.55%). In contrast, the average level of α-linolenic acid was much lower in rowanberry pomace samples (3.36%) than in chokeberry, black currant, and apple pomace (29.78%, 15.17%, and 8.45%). Only the α-linolenic acid values of two rowanberry hybrid cvs ‘Granatnaja’ (5.09%) and ‘Alaja Krupnaja’ (6.08%) reached near the value of strawberry pomace (5.55%) [30]. Moreover, the lowest linoleic acid (n-6) to α-linolenic acid (n-3) PUFA ratios were found in hybrid cvs ‘Granatnaja’ (8.34%) and ‘Alaja Krupnaja’ (6.70%), while all the other cvs had a ratio over 12 (Table 2). Western diets are characterized by high consumption of n-6, raising the 6n/3n ratio to a range of 10:1 to 20:1, which increases the risk of obesity and cardiovascular disease [35]. However, the optimal healthy balance of n6/n3 ratio should range between 1:1 to 5:1 [36]. Moreover, the World Health Organization has claimed that replacing animal based saturated fatty acids (SFA) with plant based polyunsaturated fatty acids (PUFA) decreases LDL cholesterol concentration and the total/HDL cholesterol ratio, which in turn decreases the risk of coronary heart disease (CHD) [37]. Consequently, if rowanberry pomace powder is used in food applications, other ingredients that balance the high n6/n3 ratio (>5:1) should be used together with rowanberry pomace powder. For instance, Pieszka et al. found the lowest n6/n3 ratios among fruits in chokeberry and black currant pomace, 1.45 and 2.61, respectively [30], and Grajzer et al. calculated a ratio of 4:82 in raspberry seed oil [13]. However, Radočaj et al. found an even lower ratio in raspberry seed oil (1.86) and a ratio of 4.45 in blackberry seed oil [17]. Their study also revealed that the composition of fruit pomace seed oil remains stable, despite long-term frozen storage, and can be used as a valuable raw material for oil extraction [17]. According to the findings in the current study, rowanberry seed oil especially from the cvs ‘Sahharnaja’, ‘Krasnaja’, ‘Oranzevaja’, and ‘Kubovaja’, is a considerable source of linoleic fatty acid. Therefore, these cvs can be chosen for oil extraction and for further application in food and cosmetics.

## 3. Materials and Methods

### 3.1. Preparation of Rowanberry Pomace Samples

Rowanberry cvs were harvested at the beginning of September 2019 from Polli Horticultural Research Centre, (South Estonia, 58°7′ N, 25°33′ E) and were subsequently treated as described in [38]. Briefly, immediately after picking, the fruits were frozen and stored at −20 °C in zip lock bags in batches of 4–6 kg. Juice was pressed from the thawed fruit using a low-speed juicer Smeg SJF01CREU (Smeg S.p. A, Guastalla, Italy). The pomace parts of all cvs were lyophilized in a VirTis Advantage Plus Benchtop Freeze Dryer Model XL-70 (SP Industries, Warminster, PA, USA) for 72 h at 30 µbar. The pomace samples were ground in a Retsch cutting mill (Retsch SM 300, Retsch GmbH, Haan, Germany) with sieve hole diameters of 5 mm to obtain homogenous samples for analyses. All the samples per cultivar were from one lot. 

### 3.2. Chemicals and Reagents

Sulfuric acid (H_2_SO_4_), boric acid (H_3_BO_3_), and hydrogen chloride (HCl; 0.561 M) originated from Sigma-Aldrich Chemie (Steinheim, Germany). Sodium hydroxide (NaOH, 50%) was purchased from Ingle AS (Ingliste, Estonia), and Kjeltabs (FOSS Analytical A/S, Hillerød, Denmark) tablets were from Oridor Eesti OÜ (Tartu, Estonia). Ethanol (95% and 78%) was obtained from Estonian Spirit OÜ (Tallinn, Estonia). Heat-stable α-amylase, amyloglucosidase, protease, pectate Lyase (*Aspergillus* sp.), analytical grade Celite, Tris-HCl, and MES/TRIS buffer were purchased from Megazyme Ltd. (Wicklow, Ireland). 

### 3.3. Evaluation of Proximate Composition and Pectin Content of Rowanberry Pomace

The proximate chemical composition of 16 ground rowanberry pomace samples was determined using standard AOAC methods. The moisture content was determined by drying 2–3 g of samples at 105 °C to a constant weight, according to the Association of Official Analytical Collaboration (AOAC) Method 920.39. The constant dry weight of the pomace was subtracted from the initial weight, and the obtained water content was divided by the initial weight of the sample. The ash content was determined by charring 2 g of pomace for 30 min, followed by incineration in a muffle furnace at 525 °C for 4 h, according to the AOAC Method 930.05. The residues obtained after incineration were recorded and divided by the initial weight of the sample. The crude protein content was determined using the Kjeldahl procedure (N × 6.25), according to the AOAC Method 978.04. The crude fat content was analyzed according to the AOAC Method 948.22, by extracting the fat from the sample using petroleum ether as a solvent in a Soxhlet apparatus. After the extraction, the residues were dried in an oven at 105 °C to a constant weight. The lipid content was calculated from the initial dry weight of pomace by subtracting the final weight of the residues, then dividing by the initial weight of the sample. Crude fiber content was determined as a loss on ignition of dried residue remaining after digestion of the sample with 1.25% H_2_SO_4_ (*w*/*v*) and 1.25% NaOH (*w*/*v*), analyzed by AOAC official method 978.10. The total dietary fiber (TDF) was determined according to the enzymatic-gravimetric method using a total dietary fiber kit (Megazyme, Ireland) based on AOAC 991.43 [39]. Briefly, the samples were cooked at ~100 °C with heat-stable α-amylase to achieve gelatinization, hydrolysis, and depolymerization of starch; incubated at 60 °C with protease (to solubilize and depolymerize proteins) and amyloglucosidase (to hydrolyze starch fragments to glucose); and treated with four volumes of ethanol to precipitate soluble fiber and remove depolymerized protein and glucose (from starch). The residue is filtered, washed with 95% ethanol and acetone, then dried and weighed. Pectin content was determined according to the Megazyme Pectin Identification, Assay procedure (K-PECID II/II) (Megazyme, Bray, Ireland) [40]. Briefly, pectin was dissolved in acidified (pH 2.0) deionized water and adjusted to pH 12.0 to catalyze demethylation with the production of polygalacturonic acid regions in the polymer, i.e., conversion of pectin to pectate. The pectate was incubated with pectate lyase at pH 8.0 for 30 min to cleave the polygalacturonic acid, releasing unsaturated oligosaccharides, whose absorbance values were measured at 235 nm. All extractions and technical replicates were performed in triplicate, and the results were expressed as a percentage of the dry weight of the sample.

### 3.4. Determination of Fatty Acids

**Figure 3:**
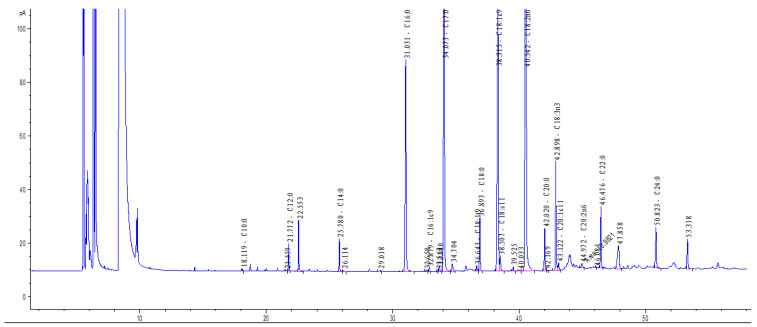
GC chromatogram of rowanberry pomace fatty acids.

Fatty acid composition in rowanberry pomace samples was determined according to the method of Sukhija and Palmquist [41] with slight modifications. Briefly, the lipids of the ground samples were extracted and methylated by applying a solvent mixture consisting of methanol, toluene, and acetyl chloride (20:27:3) for 500 mg of sample containing 3 mg internal standard (C17:0 fatty acid). The contents of fatty acid methyl esters were determined on an Agilent 6890A GC (Santa Clara, CA, USA) equipped with a split/splitless injector and a flame ionization detector, and the fatty acid methyl esters were separated on a quartz capillary column with a liquid-phase CP-Sil 88 (100 m × 0.25 mm, liquid-phase layer thickness 0.20 μm). Identification of common fatty acids were accomplished by comparison of sample peak retention times (Figure 3) with those of fatty acid methyl ester standard mixtures (Supelco 37 Component FAMEMix, Bellefonte, PA, USA, Nu-Chek Prep GLC 603, 408, BAME Mix, Linoleic Acid Methyl Ester Isomer Mix and many individual FA methyl ester standards). Theoretical response factors [42] were used to correct peak areas for individual fatty acids. Two different samples from the same brand (different lots) were analyzed in different runs at different times. All individual samples were analyzed in duplicate within the same run, from extraction to detection. Results were expressed as % of total fatty acids, which are calculated as the peak area of each fatty acid divided by the total peak area (of all fatty acids) multiplied by 100%. In order to express the results as % per sample, the peak area of the 3 mg internal standard (C17:0) was used for calculation. 

### 3.5. Statistical Analysis

The mean values and standard deviations (SD) of chemical contents (moisture, ash, protein, crude fat, TDF and CF, pectins, micro- and macro-elements and fatty acids) of pomace samples of 16 rowanberry cvs and wild rowanberry were calculated using MS Excel 2016 and one-way analysis of variance (ANOVA) at *p* value < 0.05. 

## 4. Conclusions

The by-products rich in multiple valuable compounds are an emerging topic in connection to the valorization agro-industrial waste. The current study focused on analyzing the pomace samples of 16 rowanberry cultivars, including 5 hybrid cultivars. The findings showed that all the residue samples had a high fiber content (up to 67.17% DM) and are therefore suitable as fiber-rich additives in the food industry. Thanks to its considerable pectin content, especially in hybrid cvs ‘Granatnaja’ (7.98% DM) and cv ‘Alaja Krupnaja’ (8.39% DM), rowanberry pomace can be a valuable thickener in the confectionery and jam industry. From a nutritional viewpoint, rowanberry pomace provides important benefits to food due to its valuable macro- and microelements, such as iron and potassium. Moreover, the unsaturated fatty acids in rowanberry pomace can add extra value to cosmetic and food applications. In addition to the valuable composition of rowanberry pomace, another important aspect is the environmental benefit of valorizing agro-industrial by-products, contributing to a more sustainable food chain. However, there still exist some safety-related limitations in terms of sufficiently fast processing (drying and freezing) to avoid microbial spoilage and peroxidation of berry pomace. 

Future perspectives should focus on the technofunctional properties, such as water and oil absorption, swelling, and foaming capacity, of rowanberry pomace, which will affect the rheological, textural, and sensory properties, as well as the nutritional value of the food formulations where the pomace will be used. 

## Figures and Tables

**Table 1:** Basic chemical composition of pomace samples of 16 rowanberry cultivars.

Sample	Moisture (%)	Ash (%)	Protein (%)	Crude Fat (%)	Total Dietary Fiber %	Crude Fiber %	Pectin %
‘Krasnaja’	2.65 ± 0.29 ^a^	0.13 ± 0.00 ^b^	6.96 ± 0.31 ^a^	6.26 ± 0.53 ^b^	58.74 ± 0.52 ^i^	13.71 ± 0.17 ^h^	5.52 ± 0.42 ^c^
‘Vefed’	2.18 ± 0.36 ^b^	0.10 ± 0.01 ^b^	6.49 ± 0.24 ^b^	5.45 ± 0.26 ^bc^	53.59 ± 0.86 ^l^	13.67 ± 0.23 ^h^	6.65 ± 0.62 ^b^
‘Bussinka’	3.21 ± 0.53 ^a^	0.10 ± 0.01 ^b^	3.69 ± 0.31 ^d^	5.15 ± 0.34 ^c^	54.37 ± 0.98 ^k^	13.54 ± 0.15 ^i^	7.36 ± 0.34 ^b^
‘Likernaja’	1.47 ± 0.19 ^b^	0.06 ± 0.00 ^c^	3.69 ± 0.29 ^d^	5.39 ± 0.47 ^c^	67.17 ± 0.43 ^a^	21.19 ± 0.18 ^a^	7.46 ± 0.53 ^a^
‘Angri’	2.39 ± 056 ^a^	2.70 ± 0.01 ^a^	3.92 ± 0.32 ^d^	6.14 ± 0.42 ^b^	57.98 ± 0.35 ^j^	11.38 ± 0.06 ^m^	6.47 ± 0.83 ^b^
‘Oranzevaja’	3.02 ± 0.45 ^a^	0.11 ± 0.02 ^b^	5.11 ± 0.23 ^c^	7.47 ± 0.52 ^a^	52.71 ± 0.89 ^m^	15.26 ± 0.24 ^g^	6.94 ± 0.72 ^b^
‘Rossica’	2.49 ± 0.82 ^a^	0.06 ± 0.01 ^c^	4.32 ± 0.22 ^d^	5.38 ± 0.32 ^c^	61.87 ± 1.58 ^f^	13.36 ± 0.15 ^j^	6.13 ± 0.98 ^c^
‘Rubinovaja’	2.10 ± 1.03 ^b^	0.07 ± 0.00 ^c^	7.58 ± 0.34 ^a^	5.52 ± 0.38 ^b^	65.66 ± 1.05 ^b^	17.56 ± 0.34 ^c^	7.36 ± 0.78 ^b^
‘Solnechnaja’	2.30 ± 0.81 ^a^	0.10 ± 0.00 ^b^	4.17 ± 0.32 ^d^	5.51 ± 0.46 ^b^	58.68 ± 0.86 ^h^	12.21 ± 0.42 ^l^	6.85 ± 0.58 ^b^
‘Sahharnaja’	2.74 ± 0.35 ^a^	0.09 ± 0.01 ^bc^	3.70 ± 0.52 ^d^	7.24 ± 0.55 ^a^	60.36 ± 1.87 ^g^	16.93 ± 0.06 ^d^	5.38 ± 0.32 ^cd^
‘Moravica’	2.52 ± 0.45 ^a^	0.11 ± 0.02 ^b^	3.33 ± 0.51 ^e^	6.26 ± 0.54 ^b^	62.99 ± 1.19 ^e^	15.37 ± 0.13 ^f^	7.97 ± 0.46 ^a^
‘Burka’	2.75 ± 0.42 ^a^	0.07 ± 0.01 ^c^	4.12 ± 0.62 ^d^	4.71 ± 0.22 ^c^	64.52 ± 0.62 ^c^	18.05 ± 0.31 ^b^	6.44 ± 0.22 ^b^
‘Granatnaja’	1.83 ± 0.78 ^b^	0.11 ± 0.01 ^b^	2.44 ± 0.3 ^f^	3.96 ± 0.31 ^d^	63.04 ± 1.04 ^d^	21.22 ± 0.22 ^a^	8.00 ± 0.32 ^a^
‘Rosina’	2.49 ± 0.82 ^a^	0.12 ± 0.00 ^b^	5.60 ± 0.43 ^b^	5.14 ± 0.42 ^c^	61.92 ± 0.52 ^f^	13.30 ± 0.62 ^k^	7.17 ± 0.82 ^b^
‘Kubovaja’	2.33 ± 0.80 ^a^	0.09 ± 0.00 ^bc^	4.73 ± 0.32 ^c^	7.03 ± 0.30 ^a^	64.22 ± 0.54 ^c^	16.49 ± 0.22 ^e^	5.93 ± 0.45 ^c^
‘Alaja Krupnaja’	2.84 ± 0.43 ^a^	0.08 ± 0.00 ^c^	3.67 ± 0.22 ^d^	4.2 ± 0.32 ^d^	62.66 ± 1.18 ^e^	16.94 ± 0.20 ^d^	8.39 ± 0.78 ^a^

Results are the mean values of triplicate analyses calculated in percentages (%). Different letters on columns mark significant differences at *p* < 0.05.

**Table 2:** The major fatty acid contents in the pomace samples of 16 rowanberry cvs (% of total fatty acids), % of total fatty acids per sample and n6/n3 ratio.

Rowanberry cvs	C12:0	C14:0	C16:0	C18:0	C18:1 n-9	C18:2 n-6	C18:3 n-3	C20:0	C22:0	Total Fatty Acids (%) per Sample	n6/n3
‘Burka’	0.72 ± 0.04 ^c^	1.12 ± 0.13 ^f^	16.87 ± 1.23 ^b^	3.19 ± 0.41 ^b^	16.58 ± 1.11 ^k^	50.17 ± 2.49 ^j^	4.06 ± 0.15 ^c^	1.96 ± 0.72 ^d^	1.59 ± 0.14 ^e^	2.33	12.36
‘Likernaja’	0.64 ± 0.05 ^d^	0.87 ± 0.09 ^i^	14.55 ± 1.60 ^c^	2.95 ± 0.44 ^d^	18.44 ± 1.86 ^j^	51.84 ± 4.02 ^h^	3.51 ± 0.30 ^e^	1.97 ± 0.88 ^d^	1.55 ± 0.24 ^e^	2.64	14.77
‘Granatnaja’	1.12 ± 0.09 ^b^	1.99 ± 0.40 ^b^	20.28 ± 2.29 ^a^	3.44 ± 0.79 ^a^	15.80 ± 1.80 ^l^	42.46 ± 4.98 ^l^	5.09 ± 0.21 ^b^	2.18 ± 1.37 ^b^	2.70 ± 0.31 ^c^	1.71	8.34
‘Rubinovaja’	1.10 ± 0.11 ^b^	1.63 ± 0.22 ^c^	14.77 ± 1.79 ^c^	3.09 ± 0.49 ^c^	19.55 ± 1.76 ^i^	45.18 ± 5.04 ^k^	3.71 ± 0.30 ^d^	2.27 ± 0.78 ^a^	3.44 ± 0.51 ^a^	2.99	12.18
‘Alaja Krupnaja’	1.26 ± 0.17 ^a^	2.17 ± 0.36 ^a^	20.22 ± 2.54 ^a^	3.11 ± 0.54 ^c^	15.14 ± 1.36 ^m^	40.73 ± 5.2 ^m^	6.08 ± 0.28 ^a^	2.04 ± 1.13 ^c^	2.98 ± 0.51 ^b^	1.96	6.70
‘Moravica’	0.52 ± 0.04 ^e^	1.09 ± 0.13 ^f^	10.58 ± 0.50 ^g^	2.03 ± 0.17 ^h^	23.80 ± 0.82 ^a^	52.21 ± 1.84 ^g^	3.39 ± 0.07 ^f^	1.13 ± 0.20 ^f^	1.47 ± 0.15 ^f^	3.76	15.40
‘Krasnaja’	0.53 ± 0.03 ^e^	0.88 ± 0.11 ^i^	10.17 ± 0.35 ^h^	2.13 ± 0.1 ^gf^	21.17 ± 0.32 ^f^	57.25 ± 0.95 ^b^	2.63 ± 0.05 ^i^	1.00 ± 0.20 ^h^	1.32 ± 0.14 ^f^	3.87	21.77
‘Kubovaja’	0.54 ± 0.04 ^e^	0.94 ± 0.07 ^h^	10.64 ± 0.43 ^g^	2.19 ± 0.12 ^g^	21.42 ± 0.68 ^e^	56.45 ± 1.47 ^c^	2.38 ± 0.06 ^j^	1.01 ± 0.16 ^h^	1.40 ± 0.16 ^g^	4.15	23.72
‘Oranzevaja’	0.33 ± 0.01 ^f^	0.70 ± 0.04 ^k^	11.35 ± 0.34 ^e^	1.83 ± 0.09 ^j^	20.61 ± 0.63 ^h^	57.69 ± 1.13 ^a^	2.29 ± 0.05 ^k^	0.93 ± 0.13 ^i^	1.34 ± 0.07 ^f^	4.52	25.19
‘Sahharnaja’	0.37 ± 0.04 ^f^	0.77 ± 0.17 ^j^	9.98 ± 0.52 ^i^	1.92 ± 0.25 ^i^	21.37 ± 0.49 ^e^	57.33 ± 1.36 ^b^	2.62 ± 0.03 ^i^	0.89 ± 0.25 ^j^	1.39 ± 0.07 ^g^	4.69	21.88
‘Vefed’	0.76 ± 0.07 ^c^	1.04 ± 0.12 ^g^	12.11 ± 1.02 ^d^	2.11 ± 0.26 ^f^	20.94 ± 1.90 ^g^	53.58 ± 2.96 ^f^	3.76 ± 0.09 ^d^	1.07 ± 0.85 ^g^	1.15 ± 0.13 ^h^	2.91	14.25
‘Rossica’	0.63 ± 0.01 ^d^	1.05 ± 0.07 ^fg^	11.46 ± 0.56 ^e^	2.42 ± 0.21 ^e^	21.62 ± 1.17 ^d^	54.23 ± 2.15 ^d^	2.70 ± 0.09 ^h^	1.15 ± 0.34 ^f^	1.51 ± 0.12 ^ef^	3.47	20.09
‘Solnechnaja’	0.58 ± 0.04 ^de^	1.35 ± 0.14 ^d^	10.95 ± 0.73 ^f^	1.86 ± 0.21 ^j^	22.51 ± 1.39 ^c^	53.87 ± 2.56 ^e^	3.23 ± 0.07 ^g^	0.96 ± 0.39 ^hi^	1.36 ± 0.13 ^g^	3.57	16.68
‘Angri’	0.58 ± 0.02 ^de^	0.93 ± 0.06 ^h^	11.25 ± 0.67 ^e^	2.31 ± 0.19 ^f^	23.07 ± 1.47 ^b^	53.81 ± 2.52 ^e^	2.15 ± 0.09 ^l^	1.12 ± 0.37 ^f^	1.57 ± 0.19 ^e^	3.80	25.03
‘Bussinca’	0.35 ± 0.02 ^f^	0.78 ± 0.05 ^j^	11.14 ± 0.53 ^e^	1.71 ± 0.15 ^k^	23.04 ± 1.05 ^b^	53.66 ± 2.14 ^f^	2.61 ± 0.10 ^i^	1.02 ± 0.28 ^h^	1.38 ± 0.18 ^g^	3.41	20.56
‘Rosina’	0.61 ± 0.03 ^d^	1.18 ± 0.08 ^e^	11.01 ± 0.76 ^ef^	2.15 ± 0.21 ^g^	23.79 ± 1.34 ^a^	50.56 ± 2.54 ^i^	3.57 ± 0.12 ^e^	1.24 ± 0.54 ^e^	1.68 ± 0.19 ^d^	3.09	14.16

Results are the mean values of four replicate analyses calculated in percentages (%). Different letters on columns mark significant differences at *p* < 0.05.

## Data Availability

All data generated during this study are included in this article.
